# Supplementary figures and images for: The homeodomain factor Gbx1 is required for locomotion and cell specification in the dorsal spinal cord
Source: PeerJ. 2013 Aug 29;1:e142. doi: 10.7717/peerj.142 (PMC3757465; doi:10.7717/peerj.142)

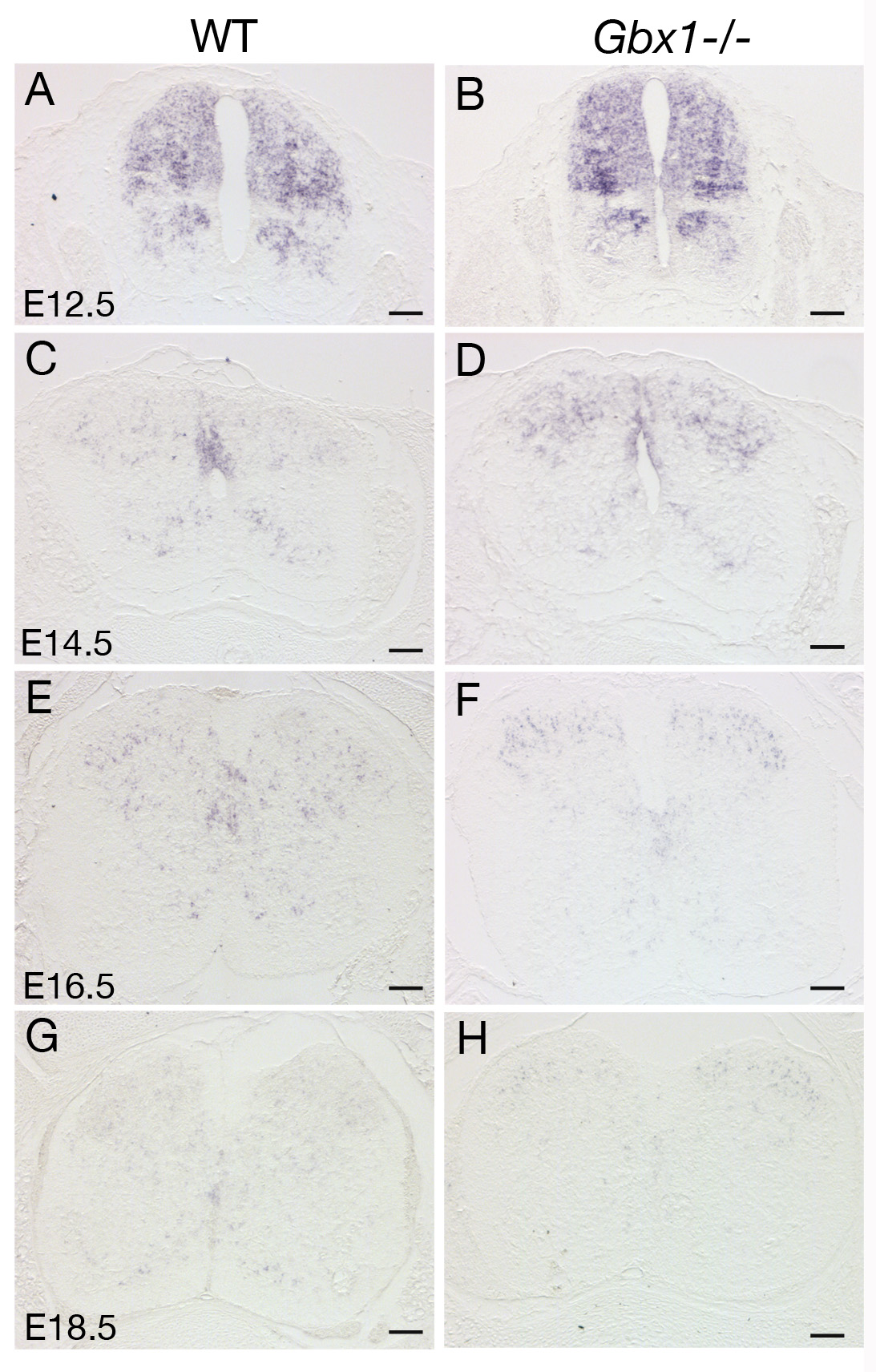

Supplement: Figure S1 — Sections through the spinal cord of wild-type (A, C, E, G) and Gbx1−/− (B, D, F, H) mice are shown. All sections are at the lumbar level. In situ hybridizations for Gbx2 were performed at different developmental stages: E12.5 (A, B; n = 2), E14.5 (C, D; n = 2), E16.5 (E, F; n = 3), and E18.5 (G, H; n = 3). Scale bars: 100 µm. [file peerj-01-142-s001.jpg]

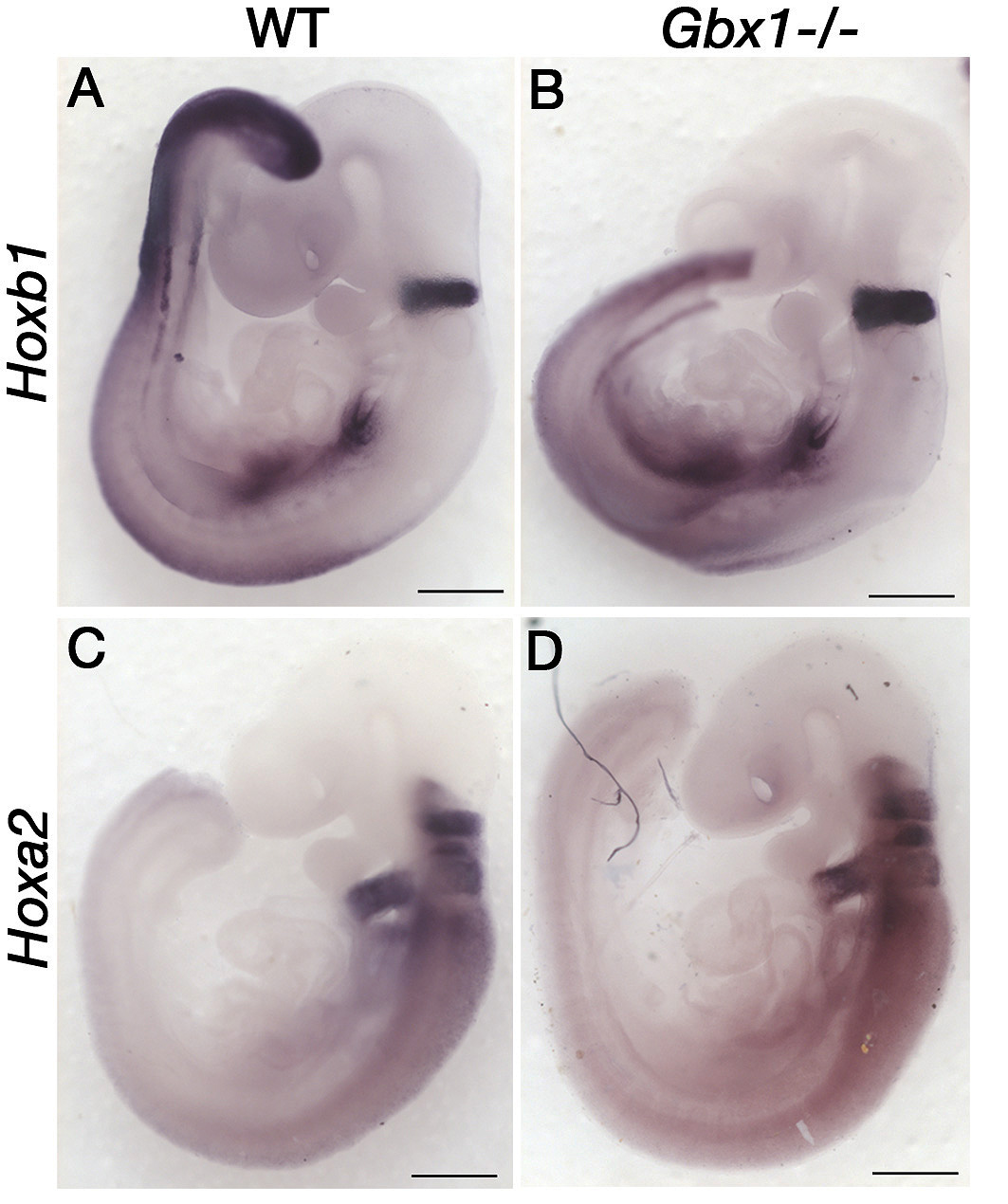

Supplement: Figure S2 — Whole-mount in situ hybridizations of E9.5 embryos with 2 markers of prospective rhombomeres: Hoxb1, which labels rhombomere 4 (A, B; n = 3), and Hoxa2, which marks rhombomeres 2 to 6 and associated neural crest (C, D; n = 3). Scale bars: 50 µm. [file peerj-01-142-s002.jpg]

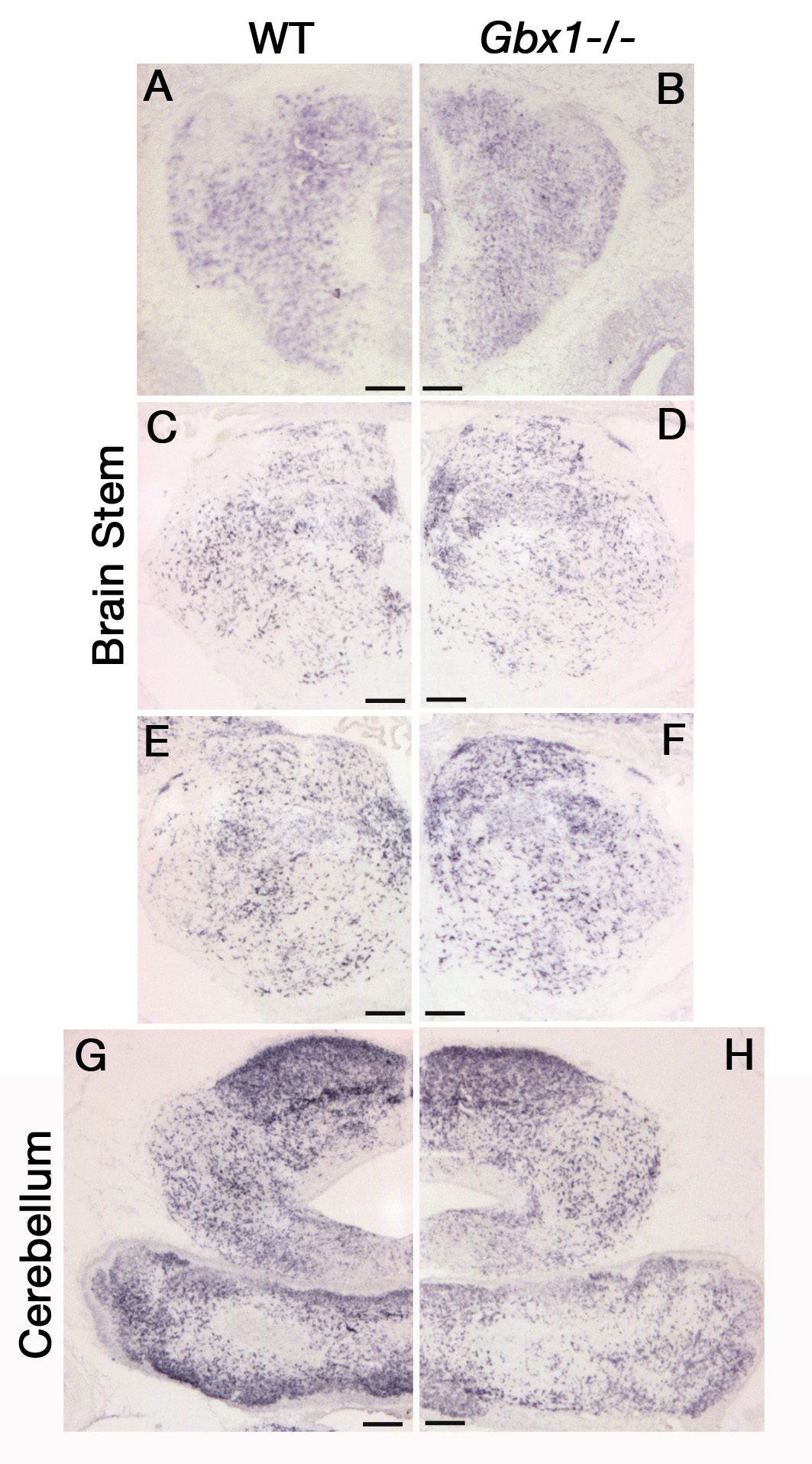

Supplement: Figure S3 — Sections are shown at various levels of the brain stem (A–F) and cerebellum (G, H) of wild-type (A, C, E, G; n = 3) and Gbx1−/− (B, D, F, H; n = 3) mice at E18.5. Scale bars: 100 µm. [file peerj-01-142-s003.jpg]

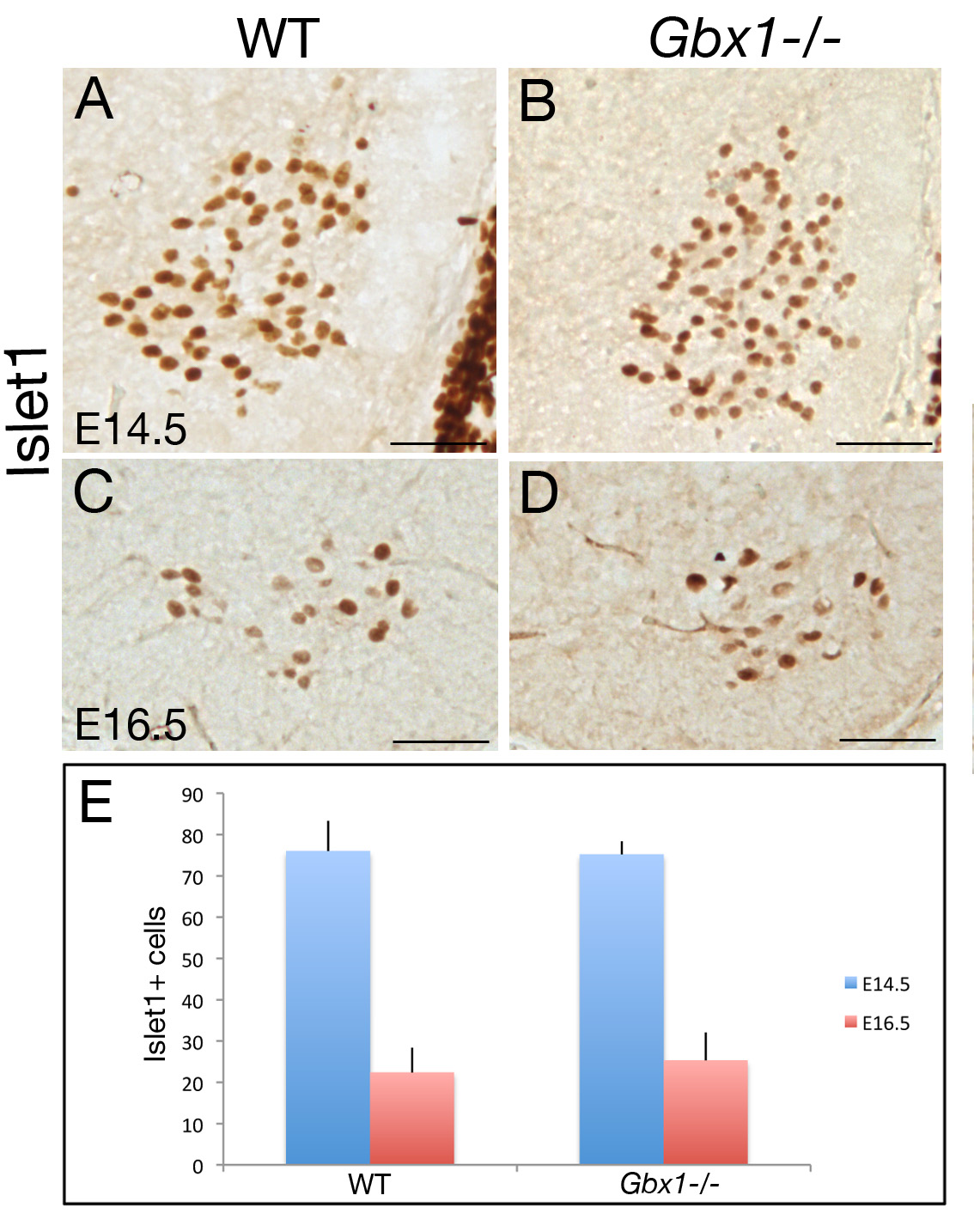

Supplement: Figure S4 — Expression of Islet1 in the lumbar spinal cord of wild-type (A, C) and Gbx1−/− (B, D) mice at E14.5 (A, B; n = 3) and E16.5 (C, D; n = 3). (E) Countings revealed that the numbers of Islet1 + cells in the ventral horn are not significantly diminished in Gbx1−/− mice (at E14.5: 76 ± 7.33 Islet1+ cells in WT; 75.22 ± 3.13 in Gbx1−/− mice; Genotype F(1, 4) = 0.27, NS, Sections F(2, 8) = 0.18, NS, Genotype*Sections F(2, 8) = 0.27, NS; at E16.5: 22.38 ± 5.96 Islet1 + cells in WT; 25.33 ± 6.70 in Gbx1-/- mice; Genotype F(1, 4) = 3.03, NS, Sections F(2, 8) = 4.73, p < 0.05, Genotype∗ Sections F(2, 8) = 5.46, p < 0.05). Scale bars: 100 µm. [file peerj-01-142-s004.jpg]
